# Selecting and defining indicators for diabetes surveillance in Germany

**DOI:** 10.17886/RKI-GBE-2018-063

**Published:** 2018-06-06

**Authors:** Lars Gabrys, Christin Heidemann, Christian Schmidt, Jens Baumert, Andrea Teti, Yong Du, Rebecca Paprott, Thomas Ziese, Winfried Banzer, Michael Böhme, Brigitte Borrmann, Reinhard Busse, Michael Freitag, Bernd Hagen, Reinhard Holl, Andrea Icks, Matthias Kaltheuner, Klaus Koch, Stefanie Kümmel, Joseph Kuhn, Oliver Kuß, Gunter Laux, Ingrid Schubert, Joachim Szecsenyi, Til Uebel, Daniela Zahn, Christa Scheidt-Nave

**Affiliations:** 1 Robert Koch Institute, Berlin; 2 University of Vechta; 3 Goethe University Frankfurt; 4 Baden-Wuerttemberg Centre for Health, Stuttgart; 5 North Rhine-Westphalian Centre for Health, Bochum; 6 Technical University of Berlin; 7 Carl von Ossietzky University of Oldenburg; 8 Central Research Institute of Ambulatory Health Care in Germany, Cologne; 9 Ulm University; 10 Heinrich Heine University Düsseldorf; 11 German Diabetes Center Düsseldorf; 12 German Center for Diabetes Research, Neuherberg; 13 Institute of licensed diabetologists, Leverkusen; 14 Institute of Quality and Efficiency in Health Care, Cologne; 15 Institute for Applied Quality Improvement and Research in Health Care, Göttingen; 16 Bavarian Health and Food Safety Authority, Oberschleißheim; 17 Institute for Biometrics and Epidemiology at the German Diabetes Center, Düsseldorf; 18 Heidelberg University; 19 University of Cologne; 20 German College of General Practitioners and Family Physicians, Berlin; 21 Federal Centre for Health Education, Cologne

**Keywords:** PUBLIC HEALTH, SURVEILLANCE, DIABETES MELLITUS, INDICATORS, NCD

## Abstract

Mainly because of the large number of people affected and associated significant health policy implications, the Robert Koch Institute (RKI) is developing a public health surveillance system using diabetes as an example. In a first step to ensure long-term and comparable data collection and establish efficient surveillance structures, the RKI has defined a set of relevant indicators for diabetes surveillance. An extensive review of the available literature followed by a structured process of consensus provided the basis for a harmonised set of 30 core and 10 supplementary indicators. They correspond to the following four fields of activity: (1) reducing diabetes risk, (2) improving diabetes early detection and treatment, (3) reducing diabetes complications, (4) reducing the disease burden and overall costs of the disease. In future, in addition to the primary data provided by RKI health monitoring diabetes surveillance needs to also consider the results from secondary data sources. Currently, barriers to accessing this data remain, which will have to be overcome, and gaps in the data closed. The RKI intentends to continuously update this set of indicators and at some point apply it also to further chronic diseases with high public health relevance.

## 1. Background

Public health surveillance is the systematic collection and analysis of relevant health data for the provision of up-to-date information and therefore provides an important basis for decisions by diverse (health) political stakeholders to protect and improve the health of the population [1-3]. Surveillance consequently is one of the key fields of public health [4] and is no longer limited merely to infectious diseases. In the 21st century, preventing chronic, noncommunicable diseases (NCD) and providing care to patients has become one of the great health challenges globally [5-7]. Cardiovascular diseases, cancer, chronic lung diseases and diabetes mellitus [8] are now ailments faced not only by wealthy countries, but increasingly and significantly also contribute to premature mortality in low and middle income countries, which has caused the World Health Organization (WHO) to define clear goals in its Global Action Plan 2013-2020 on NCD prevention and control.

Diabetes mellitus in particular has become a serious issue for healthcare systems globally [9-11]. Due to high incidence rates and the great potential offered by prevention measures, type 2 diabetes is currently a focus. The known type 2 diabetes risk factors specifically include obesity, lack of physical activity, unhealthy diet, smoking, stress and social deprivation [12]. However, the role played by environmental and psycho-social factors in type 2 diabetes is still not fully understood [13, 14]. Equally, the complex interactions between inherited and acquired risk factors over the course of a person’s life necessitate further research. During gestation and infancy epigenetic mechanisms possibly leave their mark on metabolic processes later in life [15]. Gestational diabetes potentially plays a particularly important role here. Although in most cases women recover from this form of diabetes after pregnancy, the disease is related to a greater risk of complications during pregnancy as well as an increased risk for both the mother and the baby to develop type 2 diabetes at a later stage in life [16-18].

Importantly, the life-style related risk factors for type 2 diabetes also play a key role in other important NCDs [5, 19]. Often, the large socio-economic implications of type 2 diabetes mean that the great public health relevance of type 1 diabetes, which is a far rarer form and an autoimmune disease, is also often overlooked. Mostly, this form develops at childhood or adolescent age and requires patients to take insulin for the rest of their life, with a correspondingly severe impact on their quality of life. Moreover, over the past decade a so far unexplained global increase in the number of new cases of type 1 diabetes is recognised [20, 21].

According to data from the Robert Koch Institute (RKI), an estimated 6.7 million adults in Germany are affected by diagnosed or undiagnosed diabetes [22, 23]. For adults in Germany, the data from national health monitoring suggests that around one in five cases of diabetes goes unrecognised, with a significant decrease in this figure being registered since the end of the 1990s [23]. Notwithstanding the advances in early detection and treatment, diabetes mellitus continues to entail severe complications in many patients mainly due to the damages to small and large blood vessels, as well as the peripheral and autonomic nervous system. Cardiac insufficiency, infarction and stroke, diabetic foot and amputations of the lower extremities, diabetic eye diseases and blindness, renal insufficiency and dialysis, as well as diabetic neuropathy are among the most frequent complications of diabetes mellitus. Furthermore, in women, diabetes increases the risk for complications during pregnancy [24], as well as for depression and possibly dementia [25] and is associated with an increased risk for certain types of cancer [26]. Overall mortality for adults with diabetes remains significantly higher than for people of the same age without diabetes, although the diabetes-related excess mortality varies depending on age and gender [9, 27, 28].

This explains the central role played by diabetes mellitus as an NCD [19] and why the RKI, on behalf of the Federal Ministry of Health, chose diabetes as a model disease for developing an NCD public health surveillance. Public health surveillance [19] requires a scientific framework concept with defined goals and fields of activity, evidence-based and health politically relevant indicators [29-32], as well as an integration into the established health information systems [33]. This has not yet been achieved in Germany. While the indicator set of the federal states' health reporting [34] by default includes indicators to account for diabetes-related hospital admissions, early retirements and deaths, not all federal states so far have implemented this set of indicators. Some states have additional indicators that cover, for example, outpatient care. Moreover, some federal states have already published reports on diabetes [35]. No federal state has so far established a global concept for diabetes surveillance [36]. Besides reviewing, analysing and gathering available sources of data, establishing diabetes surveillance in Germany will depend primarily on developing a corresponding scientific framework concept and selecting suitable indicators [37].

Following Germany’s 2003 national health target Diabetes mellitus type 2 [38, 39], four fields of activity were identified: (1) reducing diabetes risk, (2) improving diabetes early detection and treatment, (3) reducing diabetes complications, (4) reducing the disease burden and overall costs of the disease [37]. The focus of this article is the methodological approach, which the RKI applied to select and define its set of indicators for diabetes surveillance in Germany.

## 2. Methods

The selection and definition of indicators relevant to health policy for diabetes was a multi-stage process, which was based on two independent reviews of the literature, as well as a structured consensus process that involved a national and international level interdisciplinary panel of experts (Annex Table 1).

### 2.1 Review of the literature

#### Diabetes mellitus and NCD indicators

Regarding diabetes mellitus and NCDs, the RKI conducted a selective review of publications and information systems at the population level between January and June 2016. The search covered the current international health indicator systems of the Organisation for Economic Cooperation and Development (OECD), the European Union (European Community Health Indicators and Monitoring, ECHI) and the World Health Organization (WHO) [30, 32, 40]. A further focus were indicator-based scientific publications, reports and online information systems on diabetes mellitus and NCDs in OECD member states. The search considered publications regardless of date of publication, but was limited to publications and information in either German or English. For Germany, the health reporting, prevention, rehabilitation and social medicine working group of the Permanent Working Group of the Highest State Health Authorities (AOLG) in addition identified indicator-based, federal state level reports and publications on diabetes mellitus. Furthermore, a working group of experts from Baden-Württemberg proposed an initial indicator set [41]. Moreover, current national and selected international diabetes mellitus treatment guidelines [42, 43], cardiovascular disease prevention guidelines [44], as well as the quality targets of the type 1 and type 2 diabetes disease management programmes (DMP) were reviewed [45].

The scope of indicators considered was limited per definition to: (1) indicators on type 1 and type 2 diabetes, as well as gestational diabetes, (2) indicators related to one of the four defined fields of diabetes surveillance in Germany, (3) indicators defined in either German or English.

Indicators were excluded if they fell into one of the following categories: (1) indicators for rare forms of diabetes mellitus, (2) indicators for only specific target groups (such as patients with particular accompanying diseases), (3) and indicators that are not fully compatible with Germany’s healthcare system.

#### Quality indicators for type 2 diabetes care

A joint scientific cooperation project on diabetes surveillance [46], which was guided by the Institute for Applied Quality Improvement and Research in Health Care GmbH (aQua) in Göttingen, conducted an additional systematic review of the literature with a focus on type 2 diabetes and quality of care.

This review was conducted in the literature databases of Embase, Medline and the Cochrane Library, as well as in the indicator databases of the US Agency for Healthcare Research and Quality (AHRQ) and the British Health and Social Care Information Centre (HSCIC). Further international institutions and agencies that develop, compile or publish indicators were also reviewed. To take account of the German context, a partially automated text search was conducted of Germany’s national guidelines (NVL) on diabetes treatment [43].

The following inclusion criteria were defined: (1) indicators for adults with type 2 diabetes, (2) indicators defined in either German or English.

The criteria to exclude indicators were defined as: (1) indicators that are incompatible with the German health system, (2) indicators without clearly defined numerators and denominators, (3) indicators that relate exclusively to the care aspects of diabetes in certain population groups (e.g. African Americans in the USA).

### 2.2 Selecting and assessing indicators

At the RKI, the diabetes surveillance working group reviewed all of the indicators identified based on these parameters and supplemented them with indicators for: diabetes risk tests, rehabilitation, patient satisfaction, avoidable hospital admissions, screenings (gestational diabetes) and disease burden.

To ensure international compatibility, a national and international panel of experts conducted a structured assessment of the indicators provided by the RKI (Annex Table 1a); a process, which included a written assessment of indicators regarding their relevance to national diabetes surveillance. All members of the external panel of experts (n=35) received an email with an evaluation template attached to assess the relevance of indicators (essential, important, additional and negligible) that included blank boxes for further comments. The diabetes surveillance working group received back 17 assessment forms, which represents a 49% response rate. The results of this initial assessment were prepared in the run-up to an international scientific workshop at the RKI in July 2016 and then discussed and finalised during the workshop [37, 47].

The following step consisted of evaluating the health policy relevance of the selected indicators for diabetes surveillance in Germany. This was based on a two-tier Delphi method, which drew on the approach financed and started by the EU in the Joint Action on Chronic Diseases [48] programme, as well as the RAND/UCLA Appropriateness Method (RAM) [49].

**Figure 1:**
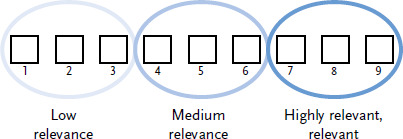
Evaluation template on indicator selection and relevance assessment Own diagram

In a first step, based on a nine-step scale, the 20 members of the diabetes surveillance scientific board (Annex Table 1b) were asked to rank all of the indicators according to their relevance for health policy. The members of the board received a corresponding assessment form as an email attachment and asked to return it to the RKI. The form permitted them to comment on the clarity, health political influenceability and international comparability of indicators. The RKI received 13 assessment forms back, which represents a 65% response rate. Subsequently these forms were evaluated and the results for each indicator comprehensively prepared:

► Indicators were classified as highly relevant if they received at least 75% from the top three grades (7-9).
► Indicators were classified as relevant if they received between 50% and under 75% of ratings from the top three grades (7-9).
► Indicators were classified as being of medium relevance if at least 50% of ratings were from the medium rates (4-6).
► Indicators were classified as being of low relevance if at least 50% of ratings were from the lowest rates (1-3) (Figure 1).

In the second step of the Delphi method, the members of the scientific board were invited to a meeting in Berlin. 16 members participated in the meeting in March 2017, which corresponds to an attendance rate of 80%. During the meeting the participating board members were presented with the results of the first assessment round and provided with the opportunity to discuss or clarify open questions or unclear definitions for each indicator. Following the discussion, all board members were asked to rate once again the indicators regarding their relevance for diabetes surveillance in writing and anonymously. The format of the assessment forms and evaluation criteria was identical to that of the first step of the procedure.

In a separate move, an additional panel of experts provided an evaluation of the indicators of the cooperation project on type 2 diabetes quality of care (Annex Table 1c). Here too, the evaluation of indicators was based on a two-tier Delphi method with a focus on type 2 diabetes quality of care. This was based on a modified RAND-UCLA appropriateness method (RAM) provided by the aQua institute [49, 50]. Relevance in this case too was assessed using the nine-step scale described in Figure 1. Both stages of the assessment procedure were conducted in writing and anonymously and included both filling out an online evaluation and an evaluation provided during a face-to-face meeting following a discussion of the results of the online evaluation.

For a final selection of indicators, the RKI evaluated the results from these review strategies. Initially, indicators from both indicator sets, which were rated as either highly relevant or relevant became part of the final selection and indicators with a medium or low relevance rating were excluded. In addition, from the indicators provided by the cooperation project those considering only specific aspects of treatment (such as amputations without vascular procedures, major amputations without a second opinion procedure) were also excluded. The remaining indicators were grouped into core and supplementary indicators for diabetes surveillance in Germany at the population level based on the following criteria:

#### Core indicators:

► Indicators classified as highly relevant, regardless as to whether they were classified as indicators for type 2 diabetes quality of care or not.
► Indicators classified as relevant, which in addition were classified as relevant for type 2 diabetes quality of care.
► Indicators classified as relevant for type 2 diabetes quality of care with a clear link to diabetes surveillance at the population level, which had so far not been included in the set of indicators used by the diabetes surveillance working group.

#### Supplementary indicators:

► Indicators classified as relevant, which were however not identified as indicators for type 2 diabetes quality of care.

The consensus-oriented closing discussion round prepared the indicators in line with the ECHI model [51] regarding the following aspects: operationalisation (definition, reference population), available stratification variables (such as age, gender and socioeconomic status), data availability, type and periodicity of appropriate data sources, scientific background, references, comments on specificities regarding definition or use. Moreover, indicators were pre-assigned to one of the four defined fields of activity in line with the 2003 national health target diabetes mellitus type 2 [38, 39].

## 3. Results

Initially, the described search strategy regarding the established diabetes and NCD reporting systems served to identify 32 indicators and/or groups of indicators. In addition, 13 indicators covering further hitherto insufficiently described fields were also adopted. This established an initial set of 45 indicators.

During the structured consensus process, in an initial evaluation round, the international panel of experts assessed 18 indicators as being ‘essential’, 14 indicators as being ‘important’, and 8 further indicators as being ‘additional’ indicators. Five indicators were rated heterogeneously, no consensus was achieved and they could therefore not be assigned to one of the categories. No indicator was classified as ‘negligible’. Furthermore, the members of the diabetes surveillance scientific board proposed four additional indicators (gestational diabetes prevalence, early retirement, hypoglycaemia incidence and information needs). They also proposed to split the indicator ‘environmental factors’ into three separate indicators (traffic exposure, walkability and social deprivation). This produced an indicator set of 51 indicators. The following national level two-tier Delphi method assessed 18 indicators as being highly relevant, 18 as being relevant and 15 as being of medium relevance. No indicator was assessed as being of low relevance.

### 3.1 Final selection of indicators

The final selection considered only indicators in the highly relevant (n=18) and relevant (n=18) groups. This step therefore excluded the 15 medium relevance indicators.

**Figure 2:**
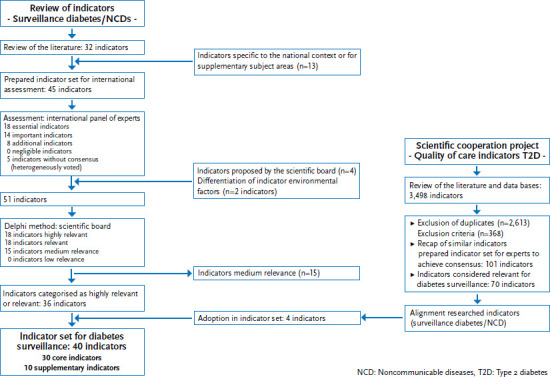
Flowchart diabetes surveillance indicator selection Own diagram

During the final step of the procedure, the remaining 36 indicators were compared to the results of the cooperation project on indicators for type 2 diabetes quality of care. Four additional indicators on relevant aspects of diabetes treatment were then added to the diabetes surveillance indicator set (age at diagnosis, renal replacement therapy, diabetic neuropathy, and diabetic foot syndrome). Moreover, core and supplementary indicators were defined based on the described criteria. The flowchart (Figure 2) provides a detailed step-by-step description of the indicators that were identified over the course of the selection process.

As a result, the resulting set of 30 core and 10 supplementary indicators/indicator groups for the four fields of activity were presented to the scientific board and approved in unanimous consensus (Table 1). The complete prepared indicator set including information on indicator operationalisation is available via the internet pages of the RKI (www.rki.de/diabsurv). Furthermore, the detailed assessments underlying the selection of indicators is included for all indicators as well as their division into core and supplementary indicators (Annex Table 2).

Where possible, and depending on the available data, the diabetes types are differentiated, and, depending on the available data, the results also stratified by age, gender, social status, education and region.

## 4. Discussion and outlook

The selected set of indicators for diabetes surveillance in Germany aims to collect detailed, comparable and successive data on diabetes mellitus in Germany. This should establish a firm basis for diabetes surveillance, and aims to ensure an up to date and comprehensive analysis of disease dynamics, potential for prevention and care needs. The epidemiologic development of incidence and prevalence will be covered, as will the development of the known risk factors, associated diseases and quality of care that can largely be influenced. What is crucial here is a differentiation by gender, age, socioeconomic status and region. This last factor establishes a bridge to federal state level health reporting.

In Germany, potentially useful and ample data is already available. As each source of data has its specific advantages and disadvantages, the described consensually agreed indicator set references different sources of data for calculating indicators depending on the availability of such data. Primary RKI health survey data for example permits an analysis stratified by socioeconomic status and further individual level aspects, and, by referring to laboratory analysis, allows estimating the prevalence of unknown diabetes in the population. Furthermore, information on a set of risk factors is collected, which facilitate predicting the development of a type 2 diabetes by means of established risk models [52]. Furthermore, population representative estimates on quality of life and a comparison of the functional limitations faced by adults with and without diabetes mellitus are possible, as are estimates on diabetes comorbidity [53] and for selected indicators on the quality of care for adults with diabetes mellitus [54]. Secondary sources of data often offer in particular the advantage of providing information on a large number of cases, the regular availability of this data and a lack of distortion effects due to non-participation. Data from statutory health insurances (GKV) for example or the associations of statutory health insurance physicians (KVen) provide up to date, periodically recurrent estimates on the development of the incidence of medically diagnosed diabetes including the possibility of differentiating between types of diabetes and region [55, 56]. An assessment of the quality of care is possible, based as much on the results of regular DMP analyses [45, 57] as on GKV routine data provided on out and inpatient treatment [58, 59].

Diabetes surveillance will therefore also need to establish the specific strengths and weaknesses of each source of data. This should also lead to recommendations regarding the use of this data and its application to other diseases for a broader surveillance of NCDs in the future. Implementation of the data transparency act (DaTraV) in particular, which was begun by the German Institute of Medical Documentation and Information (DIMDI) in 2014, meanwhile provides data for morbidity-oriented risk structure compensation (Morbi-RSA) from all statutory health insurers for research. Processing of this data constitutes a first and important step towards using routine data for surveillance. In spite of providing data for everybody insured in the GKV, the data set currently remains limited to outpatient and inpatient diagnoses and the outpatient provision of medicines. Further data on outpatient services, as well as on operations, procedures or general measures in inpatient care is not included. Certain process-oriented indicators, however, require such information. A satisfactory solution to accessing sources of data that include such information (GKV and KV routine data, as well as DMP documentation data) is still lacking. Generally, these bodies of data are only accessible in the context of research cooperation projects. Surveillance, however, would demand a regular access to such sources of data. Clearing these obstacles in a dialogue with the holders and users of such data is desirable. In addition, the indicators of quality deducted from DMP are subject to a certain degree of change over time regarding their definition and target population (definition of the numerator and denominator sample).

To define indicator sets, this process of seeking consensus seems appropriate. Establishing an interdisciplinary advisory committee has meant that indicators for relevant fields of activity in the areas of public health and prevention as well as medical treatment could be defined. Combining online assessments in writing and consensus-oriented discussion rounds during the bi-annual face-to-face board meetings is evidently a practicable and efficient approach. The established indicator set now for the first time provides a concept for a German surveillance system for one chronic, noncommunicable disease – diabetes mellitus. Initial analyses based on these newly established indicators will now begin and health reporting formats will be developed to ensure that the results are adequately prepared for the different target groups (universities, politics, patients, the general public etc.).

In the future, diabetes surveillance should serve as a model for a broader surveillance of NCDs, whereby the transferability of the model to other diseases still needs to be tested. Importantly, the indicator set should not be too rigid and developed further. This applies for example to the indicators for diabetes-related health competency in the overall population and among adults, who already have diabetes. Currently, a population representative telephone survey on disease knowledge and information needs is being conducted by the RKI in close cooperation with Germany’s Federal Centre for Health Education (BZgA). Furthermore, for a surveillance of NCDs, describing social and health policy context factors and the implementation of measures through indicators will be key. Moreover, the indicator system should be continuously further developed regarding compatibility with the nascent health information systems at the international level (WHO, EU). This applies in particular to activity field 1 (reducing diabetes risks), which will require the indicator group ‘context factors’ to be developed. Indicators in activity fields 2 and 3 (reducing diabetes complications, diabetes early detection and treatment) are defined relatively completely as regards the current quality target criteria (DMP, St Vincent criteria). Minor modifications, however, owing to the continuous updating of current definitions must be expected.

Regarding a future broader surveillance of NCDs, the goal remains to also define lead indicators analogous to the Swiss model over and above the differentiation between core and supplementary indicators which have already been established [60]. Lead indicators are defined as having an overarching relevance in terms of indicators to NCD surveillance and are collected at regular intervals and reliably in the context of a national NCD targets yet to be defined. From the current set of indicators, the epidemiological figures for prevalence, incidence and mortality, the regularly published treatment indicators of the OECD on diabetes-related amputations [30], as well as the indicators to quantify the disease burden such as disease costs or the years of life lost due to disease could serve as such lead indicators.

## Key statements

- Due to the high social relevance of diabetes and its sequelae, the Robert Koch Institute has started to establish a national diabetes surveillance system.
- Surveillance systems require indicators that are evidence-based and consented among relevant stakeholders.
- Using a multi-stage consensus process, 30 core and 10 supplementary indicators for surveillance were consented and assigned to four fields of activity.
- Data from the nationwide health monitoring of the Robert Koch Institute and routine data will be used for continuous data analysis and timely reports of results.
- The consented set of indicators is to be continously adapted and the methodological approach could be applied to other noncommunicable diseases of high public health impact.

## Figures and Tables

**Table 1:** Subject areas for indicators for diabetes surveillance in four fields of activity Own diagram

(1) Reducing diabetes risk	(2) Improving diabetes early detection and treatment
**Core indicators** Incidence Prevalence gestational diabetes Overweight/obesity Physical activity Smoking Social deprivation	**Core indicators** Prevalence of known diabetes Prevalence of unknown diabetes Disease management programme participation rate Disease management programme quality objectives achievement Quality of care Treatment profiles Health related quality of life Screening gestational diabetes Age at diagnosis
**Supplementary indicators** Prediabetes Sugary beverages consumption Absolute diabetes risk (based on the German diabetes risk test) Contextual factors	**Supplementary indicators** Check-up 35 Patient satisfaction
**(3) Reducing diabetes complications**	**(4) Reducing the disease burden and overall costs of the disease**
**Core indicators** Depression Cardiovascular diseases Diabetic retinopathy Diabetic nephropathy Renal replacement therapy Diabetic (poly-)neuropathy Diabetic foot syndrome Amputations related to diabetes Frequency of severe hypoglycaemia	**Core indicators** Direct costs Hospitalisation rate Reduced-earning-capacity pension Mortality Years of life lost Healthy life years
**Supplementary indicators** Risk of cardiovascular events Complications during pregnancy	**Supplementary indicators** Years lived with disability Disability-adjusted life years (DALYs)

## Annex Table 1 (in German) Participants Expert Committees a) International Panel of Experts

**Table:**

Prof. Dr. Winfried Banzer	Johann Wolfgang Goethe-Universität Frankfurt am Main, Institut für Sportwissenschaften
Prof. Dr. Michael Böhme	Landesgesundheitsamt Baden-Württemberg, Stuttgart
Dr. Brigitte Borrmann	Landeszentrum Gesundheit Nordrhein-Westfalen (LZG.NRW)
Dr. Ralph Brinks	Deutsches Diabetes-Zentrum (DDZ), Leibniz-Zentrum für Diabetes-Forschung an der Heinrich-Heine-Universität Düsseldorf, Institut für Biometrie und Epidemiologie
Prof. Dr. Reinhard Busse	Technische Universität Berlin, Fachgebiet Management im Gesundheitswesen
Prof. Dr. Fabrizio Carinci	University of Surrey, Guildford, School of Health Sciences
Diana Droßel	diabetesDE – Deutsche Diabetes-Hilfe
Prof. Dr. Saskia E. Drösler	Hochschule Niederrhein, University of Applied Sciences, Medizin, Medizin-Controlling und Informationssysteme
Prof. Dr. Michael Freitag	Deutsche Gesellschaft für Allgemeinmedizin (DEGAM) Carl von Ossietzky Universität Oldenburg, Department für Versorgungsforschung
Prof. Dr. Edward W. Gregg	Centers for Disease Control and Prevention (CDC), Division of Diabetes Translation
Dr. Bernd Hagen	Zentralinstitut für die kassenärztliche Versorgung in Deutschland (Zi)
Prof. Dr. Hans-Ulrich Häring	Universitätsklinikum Tübingen, Medizinische Klinik IV
Dr. Dirk Hochlenert	Deutsche Diabetes Gesellschaft
Prof. Dr. Reinhard Holl	Universität Ulm, Institut für Epidemiologie und medizinische Biometrie
Prof. Dr. Rolf Holle	Helmholtz Zentrum München, Deutsches Forschungszentrum für Gesundheit und Umwelt (GmbH), Institut für Gesundheitsökonomie und Management im Gesundheitswesen
Prof. Dr. Andrea Icks	Heinrich-Heine-Universität Düsseldorf, Medizinische Fakultät, Institut für Versorgungsforschung und Gesundheitsökonomie
Dr. Michael Jecht	Medizinisches Versorgungszentrum Havelhöhe
Prof. Dr. Jeffrey Johnson	University of Alberta, School of Public Health
Dr. Matthias Kaltheuner	Gemeinschaftspraxis Kaltheuner – v. Boxberg, Leverkusen
Dr. Klaus Koch	Institut für Qualität und Wirtschaftlichkeit im Gesundheitswesen (IQWIG), Ressort Gesundheitsinformation
Prof. Dr. Bernhard Kulzer	Diabetes Zentrum Mergentheim
Dr. Joseph Kuhn	Bayerisches Landesamt für Gesundheit und Lebensmittelsicherheit, Dienststelle Oberschleißheim
Prof. Dr. Oliver Kuß	Deutsches Diabetes-Zentrum (DDZ), Leibniz-Zentrum für Diabetes-Forschung an der Heinrich-Heine-Universität Düsseldorf, Institut für Biometrie und Epidemiologie
Prof. Dr. Gunter Laux	Universitätsklinikum Heidelberg, Abteilung Allgemeinmedizin und Versorgungsforschung
Prof. Dr. Jan Mainz	Aarhus University, Studienævnene på HE – Studies in Health Science
Dr. Wolfgang Rathmann	Deutsches Diabetes-Zentrum (DDZ), Leibniz-Zentrum für Diabetes-Forschung an der Heinrich-Heine-Universität Düsseldorf, Institut für Biometrie und Epidemiologie
Helmut Schröder	Wissenschaftliches Institut der AOK (WIdO)
Dr. Ingrid Schubert	PMV forschungsgruppe, Klinik und Poliklinik für Psychiatrie und Psychotherapie des Kindes- und Jugendalters, Universität zu Köln
Prof. Dr. Jochen Seufert	Deutsche Diabetes Gesellschaft Universitätsklinikum Freiburg, Abteilung Endokrinologie und Diabetologie
Dr. Dominik Graf von Stillfried	Zentralinstitut für die kassenärztliche Versorgung in Deutschland (Zi)
Dr. Heidrun Thaiss	Bundeszentrale für gesundheitliche Aufklärung (BZgA)
Dr. Til Uebel	Deutsche Gesellschaft für Allgemeinmedizin (DEGAM) Praxisgemeinschaft Uebel, Ittlingen
Dr. Dietmar Weber	Deutsche Diabetes Gesellschaft
Prof. Dr. Sarah Wild	Centre for Population Health Sciences, University of Edinburgh

## Annex Table 1 (in German) Participants Expert Committees b) Scientific Advisory Board

**Table:**

Prof. Dr. Winfried Banzer	Johann Wolfgang Goethe-Universität Frankfurt am Main, Institut für Sportwissenschaften
Prof. Dr. Michael Böhme	Landesgesundheitsamt Baden-Württemberg, Stuttgart
Dr. Brigitte Borrmann	Landeszentrum Gesundheit Nordrhein-Westfalen (LZG.NRW)
Prof. Dr. Reinhard Busse	Technische Universität Berlin, Fachgebiet Management im Gesundheitswesen
Diana Droßel	diabetesDE – Deutsche Diabetes-Hilfe
Prof. Dr. Michael Freitag	Deutsche Gesellschaft für Allgemeinmedizin (DEGAM) Carl von Ossietzky Universität Oldenburg, Department für Versorgungsforschung
Dr. Bernd Hagen	Zentralinstitut für die kassenärztliche Versorgung in Deutschland (Zi)
Prof. Dr. Hans-Ulrich Häring	Universitätsklinikum Tübingen, Medizinische Klinik IV
Prof. Dr. Reinhard Holl	Universität Ulm, Institut für Epidemiologie und medizinische Biometrie
Prof. Dr. Andrea Icks	Heinrich-Heine-Universität Düsseldorf, Medizinische Fakultät, Institut für Versorgungsforschung und Gesundheitsökonomie
Dr. Matthias Kaltheuner	Gemeinschaftspraxis Kaltheuner – v. Boxberg, Leverkusen
Dr. Klaus Koch	Institut für Qualität und Wirtschaftlichkeit im Gesundheitswesen (IQWIG), Ressort Gesundheitsinformation
Dr. Joseph Kuhn	Bayerisches Landesamt für Gesundheit und Lebensmittelsicherheit, Dienststelle Oberschleißheim
Prof. Dr. Oliver Kuß	Deutsches Diabetes-Zentrum (DDZ), Leibniz-Zentrum für Diabetes-Forschung an der Heinrich-Heine-Universität Düsseldorf, Institut für Biometrie und Epidemiologie
Dr. Wolfgang Rathmann	Deutsches Diabetes-Zentrum (DDZ), Leibniz-Zentrum für Diabetes-Forschung an der Heinrich-Heine-Universität Düsseldorf, Institut für Biometrie und Epidemiologie
Helmut Schröder	Wissenschaftliches Institut der AOK (WIdO)
Dr. Ingrid Schubert	PMV forschungsgruppe, Klinik und Poliklinik für Psychiatrie und Psychotherapie des Kindes- und Jugendalters, Universität zu Köln
Prof. Dr. Jochen Seufert	Deutsche Diabetes Gesellschaft Universitätsklinikum Freiburg, Abteilung Endokrinologie und Diabetologie
Dr. Heidrun Thaiss	Bundeszentrale für gesundheitliche Aufklärung (BZgA)
Dr. Til Uebel	Deutsche Gesellschaft für Allgemeinmedizin (DEGAM) Praxisgemeinschaft Uebel, Ittlingen

## Annex Table 1 (in German) Participants Expert Committees c) Expert Panel on Type 2 Diabetes Care

**Table:**

Prof. Dr. Michael Freitag	Deutsche Gesellschaft für Allgemeinmedizin (DEGAM) Carl von Ossietzky Universität Oldenburg, Department für Versorgungsforschung
Prof. Dr. Michael Böhme	Landesgesundheitsamt Baden-Württemberg, Stuttgart
Dr. Dirk Hochlenert	Deutsche Diabetes Gesellschaft
Dr. Dietmar Weber	Deutsche Diabetes Gesellschaft
Dr. Petra Kaufmann-Kolle	AQUA-Institut, Göttingen

## Annex Table 2 Template for consensus on the diabetes surveillance indicator set Own diagram

**Table:**
